# Conceptualising social media addiction: a longitudinal network analysis of social media addiction symptoms and their relationships with psychological distress in a community sample of adults

**DOI:** 10.1186/s12888-023-04985-5

**Published:** 2023-07-13

**Authors:** Deon Tullett-Prado, Jo R. Doley, Daniel Zarate, Rapson Gomez, Vasileios Stavropoulos

**Affiliations:** 1 Institute for Health and Sport, Victoria University, Melbourne, Australia; 2 RMIT, Melbourne, Australia; 3 Federation University, Ballarat, Australia; 4 National and Kapodistrian University of Athens, Athens, Greece

**Keywords:** Longitudinal network analysis, Psychological distress, Social media addiction

## Abstract

**Background:**

Problematic social media use has been identified as negatively impacting psychological and everyday functioning and has been identified as a possible behavioural addiction (social media addiction; SMA). Whether SMA can be classified as a distinct behavioural addiction has been debated within the literature, with some regarding SMA as a premature pathologisation of ordinary social media use behaviour and suggesting there is little evidence for its use as a category of clinical concern. This study aimed to understand the relationship between proposed symptoms of SMA and psychological distress and examine these over time in a longitudinal network analysis, in order better understand whether SMA warrants classification as a unique pathology unique from general distress.

**Method:**

*N* = 462 adults (*M*_age_ = 30.8, *SD*_age_ = 9.23, 69.3% males, 29% females, 1.9% other sex or gender) completed measures of social media addiction (Bergen Social Media Addiction Scale), and psychological distress (DASS-21) at two time points, twelve months apart. Data were analysed using network analysis (NA) to explore SMA symptoms and psychological distress. Specifically, NA allows to assess the ‘influence’ and pathways of influence of each symptom in the network both cross-sectionally at each time point, as well as over time.

**Results:**

SMA symptoms were found to be stable cross-sectionally over time, and were associated with, yet distinct, from, depression, anxiety and stress. The most central symptoms within the network were tolerance and mood-modification in terms of expected influence and closeness respectively. Depression symptoms appeared to have less of a formative effect on SMA symptoms than anxiety and stress.

**Conclusions:**

Our findings support the conceptualisation of SMA as a distinct construct occurring based on an underpinning network cluster of behaviours and a distinct association between SMA symptoms and distress. Further replications of these findings, however, are needed to strengthen the evidence for SMA as a unique behavioural addiction.

## Introduction

In recent years, increased attention has been paid to phenomena of excessive social media use, impacting users’ lives in a way not dissimilar to substance addiction [1]. When in this state, known as ‘Problematic Social Media Use (PSMU), one’s social media usage occupies their daily life, to the extent that their other roles and obligations maybe compromised (e.g., family, romance, employment; [1, 2]. In that line, PSMU impact has been demonstrated by its significant associations with mood disorder symptoms, low self-esteem, disrupted sleep, reduced physical health and social impairment [3, 4]. Given that PSMU prevalence has been estimated to vary globally between 5%-10% of the social media users’ population [1, 5, 6], which exceeds 80% among more developed countries, such as Australia, and has the prospective to rise [7, 8], PSMU related mental health concerns present compelling. Despite these, a rather disproportional paucity of longitudinal research regarding the nature, causes and treatment of PSMU has been repeatedly illustrated [1, 9]. Attending such remarks, the present study aspires to examine the structure of PSMU’s most popular conceptualisation (as inspired by the behavioural addiction model [2]), whilst concurrently assessing its relationship with depression/distress behaviours via adopting and innovative network approach.

### Conceptualizing problematic social media use

When attempting to conceptualise PSMU, the most employed definitions involve the so called “behavioural addiction model” [1, 9]. Labelled as ‘Social Media Addiction’ (SMA), this conceptualization of PSMU is characterized by a deep fixation/drive towards the use of social media that has become uncontrollable and unhealthy. This model features a number of addiction symptoms drawn from those experienced by substance and gambling addicts, with six symptoms derived from Griffiths key-components of addiction [10, 11]. These symptoms entail *salience* (i.e., preoccupation with social media usage), *mood modification* (i.e. using Social Media to alleviate negative moods/states), *tolerance* (i.e. requiring more social media engagement over a period of time in order to attain the same degree of satisfaction/mood modification), *withdrawal* (i.e. the experience of discomfort/distress/irritability/frustration, when attempting to cease/reduce use), *relapse* (i.e. failed attempts to control social media usage) and *conflict/social impairment* (i.e. social media use interferes with, and damages, one’s social life, emotional wellbeing, educational attainment, career and/or other activities/needs; [12]).

A number of separate theories have also been put forwards, such as models describing Problematic Social Media Use in terms of dysfunctional motivations or contexts for use [13, 14]. Similarly, various instruments have been developed to reflect conceptual variability when assessing PSMU (e.g., Social Media Disorder Scale [15]; Bergen Social Media Addiction Scale [11]). However, the SMA model, as characterized by Griffiths 6 core components of addiction has seen the most use and acceptance, with a number of studies having evidenced the manifestation of those symptoms (e.g., tolerance, relapse, conflicts [11, 16], identified motivations and risk factors similar to addiction (e.g., brain/neurological similarities between substance and SMA addicts [13, 14, 17]) and developed measurement tools based on this model [9, 11, 15, 18]. Based on the above, the six symptom SMA model of PSMU, as measured via the Bergen Social Media Addiction Scale (BSMAS [11]) is employed going forward in this study.

Despite this level of acceptance, this “addiction” like definition of PSMU/SMA remains the object of controversy [19]. Criticisms abound regarding the model, with some labelling it a premature pathologizing of ordinary social media use behaviours with low construct validity and little evidence for its existence [19, 20]. For example, Huang [21] highlight positive associations between social media and physical activity, denoting that not all social media use would necessarily represent a problematic behavior. Nonetheless, the lack of clarity surrounding the links between excessive social media use symptoms and markers of impairment, such as distress has been pointed out as cause for caution [19]. For instance, it has been argued that while preoccupation behaviours may be harmful when involving substances, they don’t necessarily carry the same weight in a behavioural addiction such as SMA [22]. In addition, it is argued that links between SMA and more well recognised disorders, such as Depression, may imply that SMA is in fact a secondary symptom of pre-existing depression, and not a distinct condition itself [19]. Given that research in this area is still highly exploratory these criticisms are difficult to dispel [9]. Thus, there is a need for research clarifying the nature of SMA, its longitudinal effects, and the relative importance of each SMA proposed symptom, as well as ways in which symptoms associate risk factors/negative outcomes.

### SMA and longitudinal network analysis

One avenue of addressing this need could be offered via the implementation of longitudinal network analysis [23]. Network analysis is an exploratory approach of assessing constructs, as mirroring networks of symptoms/behaviours, where a number of variables/behaviours are examined together, whilst information is simultaneously collected regarding their inter-relationships and relative influence, so as to create a graphical ‘network’ (i.e., visualization of the construct’s underpinning behaviours; [23–25]). This analysis allows one to examine a set of symptoms from an utterly different viewpoint than traditional latent-variable perspectives. Rather than viewing symptoms as resulting from the presence of a latent construct (SMA for example), network analysis assumes symptoms are *formative.* Which is to say, as causes in themselves, interacting with each other and with other risk factors/negative outcomes to compose/form the “disorder” [24]. This allows the unique relationships, known as “edges”, between all considered variables/behaviours/manifestations, called “nodes”, to be observed, in a capacity not available with traditional structural equation modelling (SEM [26]). For example, examination of the so called symptom “centrality” (i.e. relative influence of each distinct symptom on other symptoms/behaviours included in an examined network), instead of symptom severity, may enable the detection of symptoms/behaviours with the largest influence on others, and thus contribute in evaluating: a) their “central” (or more peripheral role) in defining a proposed disorder (e.g. SMA), and; b) their targeted priority in a potential intervention program [27]. This can be done in great detail with separate centrality indices providing an indication of: a) the summed associations between a symptom/behaviour and all others examined (i.e., strength; Expected Influence in the case of psychopathology); b) the degree to which a symptom serves as an intermediary between others (i.e. betweenness) and; c) how closely a symptom aligns with others (i.e., closeness [28]). Furthermore, similar centrality relationships between distinct clusters of symptoms can be examined, with the so called “bridge” (i.e. a point that connects two distinct groups of behaviours) centrality indices (i.e. bridge strength; bridge expected influence; bridge betweenness and closeness) providing indications of which symptoms bind distinct disorders, such as SMA and depression together, either serving as intermediaries between disorders and/or by being more proximal to other disorders [28].

Such detailed examination of the relationships between symptoms, and clusters of symptoms, can further serve to test the veracity of models and constructs, which is particularly important for solidifying the occurrence of SMA [19]. For example, if the symptoms/behaviours informing a model, don’t relate at all, or accumulate into tight, separate ‘clusters’, then the construct may not be valid [29]. Additionally, with testing identical construct networks across two or more timepoints, the over-time stability of a proposed network can be examined, further validating a given construct (i.e., if the SMA symptoms’ network remains stable over time, then the construct is likely experienced longitudinally similarly [30]).

Aside of considering the stability of a network over time, network analysis procedures enable attaining stability coefficients for the edge weights and centrality indicators irrespective of the population/data examined via the use of case-dropping bootstrapping to examine the potential variance in these indices (i.e. network analysis indices such as strength and/or expected influence are re-estimated based on various alternative compositions/ re-samples of the data considered [31, 32]. Unstable indices, either population-wise or over time are invalid, and their use is generally dismissed [33]. Finally, network analysis gives one the opportunity to evaluate not only the relationships of behaviours being considered as composing a single disorder, but also to examine how these distinct disorder informing symptoms/behaviours may interact with other separate comorbid disorders (i.e. in this case SMA behaviours and depression/ anxiety [31]). This allows the examination of how these variables formatively interact with one another, as well as indicating their separate/distinct concurrent validity [34].

Indeed, the need of securing such information regarding the distinct proposed SMA symptoms and their associations with comorbid depression and/or distress behaviours experienced is reinforced by recent item response theory (IRT) and network analysis findings of responses on the Bergen Social Media Addiction Scale [35, 36]. Stănculescu [35] identified SMA behaviours of “salience” and “withdrawal” as having the highest centrality, whilst SMA “relapse” behaviours as having the lowest centrality, in the context of the 6 SMA symptoms consisting of a single unitary cluster with strong inter-relations. However, these findings despite constituting an important step, present limited in a number of ways. Firstly, they are derived from a Romanian sample (*N* = 705), where specific cultural characteristics may apply, restricting their generalizability to different populations. Secondly, due to being cross-sectional they don’t allow the examination of the stability of the network associations over-time [29, 31, 32]. Thirdly, Stănculescu’s [35] examination of the SMA symptom network only took expected influence into account considering centrality and did not consider the significance of differences in the centrality of nodes. Finally, the network examined by Stănculescu [35] involved no covariates aside of the 6 SMA symptoms. Thus, the extent of differentiation of various SMA behaviours/criteria from comorbid conditions and/or their specific associations with other commonly proposed SMA risk factors and negative outcomes (e.g. depression, anxiety) could not be established [37]. To contribute to the available knowledge in the field, the present study aims to use network analysis modelling to longitudinally examine SMA symptoms in conjunction with commonly proposed comorbid excessive digital media usage conditions involving experiencing distress (i.e., depression and anxiety [37–39]).

### Distress and SMA

Psychological distress is defined as a state of psychological suffering characterized by anxiety, depression and stress, and often serves as a general measure of mental health [37, 40]. In this capacity, investigating the ways in which SMA and distress behaviours interact, can potentially produce a clearer understanding for how a person’s mental health could be distinctly affected by the separate symptoms of SMA and/or the vice versa (e.g., Is it SMA related preoccupation, tolerance and/or withdrawal more related to anxiety and/or depression experiences?). As distress involves some of the most well researched comorbidities of SMA (e.g., depression, anxiety), there is a wealth of prior research indicating the presence of distress-SMA interactions [41, 42]. For instance, different aspects of social media use, such as the purpose of using social media (e.g., adaptive/maladaptive coping mechanisms [43]), their preferred social media activities, as well as behaviours of excessive social media usage have been consistently associated with an individual’s proneness/risk for depression, anxiety and stress [41, 42]. Such links tend to be more evident in younger populations, where social media use often drives/underpins psychological distress for a proportion of users (e. g. a developing individual might feel distressed for deviating from what is presented as ideal or common by their peers online [44]). A wide variety of explanations have been put forth as potential reasons for such distress-SMA links involving: a) distressed individuals excessively utilizing social media use as a way to cope; b) the deleterious effects excessive social media use has on sleep, time management, physical activity, the development of social skills and; c) the near constant access social media provides to information of others, prompting comparisons and negative social interactions [42]. However, these, independent findings present as fragmented, the clinically relevant, over-time links/associations between specific SMA symptoms and the levels of depression, anxiety and stress one experiences remaining unclear. Such clinically important knowledge can be offered by longitudinal network analysis, which has not been yet, to the best of the authors’ knowledge, attempted concerning these variables.

The findings of such an analysis are envisaged to also have significant epidemiological utility. Given the acknowledged connection between psychological distress and SMA behaviours [41, 42], and the noted drive of psychologically distressed individuals towards coping strategies involving escapism via social media facilitated pleasurable activities [44], it is possible-and indeed argued by some-that PSMU may not in fact represent an addiction (the SMA model) but simply be a secondary symptom of distress [19]. By examining the SMA model in conjunction with symptoms of distress, the connections between the SMA symptoms and Distress symptoms can be demystified with detail, their bridges can be identified, whilst deeper insight may be gleaned into the relationship between Distress and SMA.

### The present study

Prompted by the above literature, the present study aimed to contribute to the field via innovatively, longitudinally, examining a normative, community sample of social media users, assessed across two time points, one year apart, regarding both their SMA and distress behaviours. Specifically, it assessed their responses via advanced longitudinal network analysis’ modelling, enhanced by the use of machine learning algorithms to increase knowledge regarding: a) the validity/sufficiency of the widely popular SMA conceptualization; b) persistent differential diagnosis considerations regarding SMA and distress conditions entailing depression, anxiety and stress and; c) pivotal/central behaviours considering SMA manifestations over time. Thus, the following three aims were devised: 1) To reveal/describe the network structure of the six SMA symptoms and symptoms of depression, anxiety and stress; 2) To examine potential clustering in this revealed SMA-distress network, as well as to identify any specific bridges or routes between the clusters in this network, and; 3) To examine the stability of the revealed SMA-distress network over time and across different potential sample compositions.

## Method

### Participants

An online sample of adult, English speaking participants aged 18 to 64 who were familiar with social media [*N* = 462, *M*_age_ = 30.8, *SD*_age_ = 9.23, *n*_males_ = 320 (69.3%), *n*_females_ = 134, (29%), *n*_other_ = 9, (1.9%); 968 complete responses wave 1- 506 attrition between waves = 462] was assessed across two time points, 12 months apart. Acknowledging that adequate sample size rules of thumb are still explored for longitudinal network analysis [45], the current sample size well exceeds the threshold of 350 recommended for sparse networks up to 20 nodes in order to accurately estimate moderate sensitivity, high specificity and likely high edge weights correlations [46]. Furthermore, the 53.27% attrition (*N* = 506) between the two waves of data collection was studied. Specifically, attrition/retention was inserted as an independent dummy coded variable (i.e. 1 = attrition, 0 = retention between wave 1 and wave 2) to assess its associations with sociodemographic characteristics of the sample (via crosstabulation, X^2^), as well with SMA, depression, anxiety and stress rates (via t test). There were no significant associations between social media scores at time-point 1 and 2 (*Welch’s t*_[953]_ = 1.60, *p* = 0.11, *Cohen’s d* = 0.10). Moreover, older straight males showed decreased attrition rates (Age: *Welch’s t*_[960]_ = -4.05, *p* < 0.01, *Cohen’s d* = -0.26; Gender: χ^2^_[2]_ = 12.4, *p* < 0.01, *Cramer’s V* = 0.11); however, all differences represented a small effect size. In terms of sociodemographic, variations were observed, with very significant amounts of our sample heralding from diverse backgrounds. For example, 38.1% of the sample heralded from non-white backgrounds and 30.5% of the sample was female or nonbinary. See Table 1 for the sociodemographic information of those addressing both waves and included in the current analyses.

**Table 1:** Socio-demographic and characteristics of participants

Sociodemographic variables 426		Male *n*	%	Female *n*	%	Non-binary /Other *n*	%
White/Caucasian		321	69.5	132	28.6	9	1.9
Ethnicity	Black/African	202	43.7	77	16.7	7	1.5
	American	17	4.7	12	2.6	1	0.2
	Asian	66	14.3	26	5.6	0	0.0
	Hispanic/Latino	15	3.2	4	0.9	0	0.0
	Other (Aboriginal, Indian, Pacific Islander, Middle eastern, Mixed, other)	21	5.8	13	2.8	1	0.2
Sexual Orientation	Heterosexual /Straight	267	57.8	92	19.9	1	0.2
	Homosexual/ Gay	20	4.3	7	1.5	1	0.2
	Bisexual	29	8.0	25	5.4	4	0.9
	Other	5	1.1	8	1.7	3	0.6
Employment status	Full Time	144	31.2	43	9.3	1	0.2
	Part Time/ Casual	44	12.2	20	4.3	1	0.2
	Self Employed	48	10.4	17	3.7	2	0.4
	Unemployed	68	18.8	33	7.1	3	0.6
	Student/Other	53	11.5	32	6.9	4	0.9
Level of Education	Elementary /Middle school	4	0.9	0	0.0	0	0.0
	High School or equivalent	76	21.0	31	6.7	5	1.1
	Vocational/ Technical School/Tafe	31	6.7	14	3.0	0	0.0
	Some Tertiary Education	55	11.9	26	5.6	2	0.4
	Bachelor’s Degree (3 years)	68	18.8	27	5.8	1	0.2
	Honours Degree or Equivalent (4 years)	46	10.0	15	3.2	1	0.2
	Masters Degree (MS)	25	5.4	9	1.9	0	0.0
	Doctoral Degree (PhD)	3	0.8	2	0.4	0	0.0
	Other/Prefer not to say	13	2.8	8	1.7	0	0.0

Percentages represent portions within anyone grouping, rather than percentages of the overall population

### Measures

Aside of collecting socio-demographic information the following instruments were employed for the current study:

#### *Bergen Social Media Addiction Scale (BSMAS; *[11]*)*

The BSMAS measures the severity of one’s experience of the six proposed SMA symptoms via an equivalent number of items that ask to which degree certain behaviours associated with these symptoms relate to one’s own life (i.e., salience, tolerance, mood modification, relapse, withdrawal and conflict [11]). The items of the BSMAS include “*You spend a lot of time thinking about social media or planning how to use it*” (salience), “You feel an urge to use social media more and more” (tolerance), “You use social media in order to forget about personal problems” (mood modification), “You have tried to cut down on the use of social media without success” (Relapse), “You become restless or troubled if you are prohibited from using social media” (withdrawal) and “You use social media so much that it has had a negative impact on your job/studies” [11]. These items are rated on a 5-point scale scored from 1 (very rarely) to 5 (very often), with higher scores indicating a greater experience of SMA Symptoms [11]. A total score ranging between 6 and 30 is comprised by the accumulation of the different items’ points reflecting overall SMA behaviors. Considering the current sample, Cronbach’s α and the McDonalds ω internal reliability indices were both 0.88 for time point one and increased to 0.90 for time point two.

#### *Depression, Anxiety and Stress Scales-1 (DASS-21; *[47]*)*

The DASS measures distress experiences and comprises 21 items, subdivided into three equal subscales (7 items each) addressing depression, anxiety and stress respectively [47]. Items examine distress behaviors with a 4-point likert-type scale ranging from 0 (did not apply) to 3 (applied most of the time). Total scores for each dimension are derived by the accumulation of the relevant items’ points ranging between 0–21 for the three factors. Considering time point 1, the Cronbach’s α indices for the subscales of depression, anxiety and stress were 0.94, 0.85 and 0.88 respectively and their corresponding McDonalds ω reliabilities were 0.94, 0.86 and 0.88. For time point 2, the same Cronbach α reliabilities were 0.93, 0.85 and 0.86 and their McDonalds ω reliabilities were 0.93, 0.86 and 0.86.

### Procedure

Approval was received from the Victoria University Human Research Ethics Committee (HRE20-169) and data for both time points was collected between 2020 and 2022. Time point 1 data (*N*_t1_ = 968) was collected via an online survey link distributed via social media (e. g. Facebook; Instagram; Twitter), digital forums (e.g., reddit) and the Victoria University learning management system. The link first took potential participants to the Plain Language Information Statement (PLIS), which informed about the study requirements, responses’ anonymity and free of penalty withdrawal rights. After completing this step, eligible participants were asked to voluntarily provide their email address to be included in prospective data collection wave(s), and to digitally sign the study consent form (box ticking). Twelve months later (between August 2021 and August 2022), follow up emails involving an identical survey link (i.e., PLIS, email provision for the second wave, consent form and survey questions) were sent out for those interested to participate in the second data collection wave (*N*_t2_ = 462). Participation in this study was voluntary.

### Statistical analyses

A network model involving the six BSMAS symptoms and three DASS subscales was estimated for the two timepoints using the *qgraph* and *networktools* R packages [32, 48]. Network models involve the creation of a network nodes and edges, where nodes represent considered variables/observations and edges the relationships between them [49]. Stronger relationships/edges are represented by thicker, darker lines with the distance between variables/nodes indicating their relevance/association (closer = higher relevance) and the colour indicating the direction of the relationship (Blue = positive, red = negative). This is done in the present case via the use of zero order correlations (i.e., no control for the influence of any other variables) combined with a graphical Least Absolute Shrinkage and Selection Operator algorithm (g-lasso; [49]) employed to shrink partial correlations to zero. Practically, this reduces the chance of false positives (i.e., Type 1 error), providing more precise judgements about the relationships between variables, whilst concurrently pruning excessively weak links to simplify networks [50].

#### Cross-sectional network stability

Once network models are estimated across time points, their respective centrality, edge weights and bridge values are assessed [49]. Centrality measures used here involve: a) degree (i.e., the number of links/edges held by each node); b) betweenness (i.e. the number of times a node lies on the shortest path between other nodes); c) closeness (i.e. the ‘closeness’ of each node to all other nodes); d) eigenvector (i.e. node centrality based not the node’s connections and additionally the centrality of the nodes they are connected with)] and; d) the ‘expected influence’ of a node for the whole network [51]. The latter accounts for negative influences/edges, promotes the overall stability in the network, and it is recommended for psychopathological networks [29]. Finally, bridge values represent the rate of nodes serving as connections between distinct network clusters and are measured via bridge expected influence indices [48].

The prerequisite for estimating these values is calculating their stability coefficients across time points. These denote the estimated maximum number of cases that can be dropped from the data to retain, with 95% probability, a correlation of at least 0.7 (default) between original network indices and those computed with less cases with an acceptable minimum probability of > 0.25 and preferably > 0.5 [32]. These were calculated using a modified version of the *bootnet* package with an end coefficient representing the proportion of the original sample that can be dropped before the centrality, bridge and edge weight values vary significantly [32].

#### Cross sectional network characteristics

Once network stability is confirmed, the *networktools* package estimates the centrality, edge weight and bridge indices and graphs the network. Judgements regarding differences in centrality across nodes or in the strength of edges are made using the centrality/edge difference tests via the bootnet R package [32]. These construct a confidence interval between the two regarded results, adjusted so that the lower the stability the greater the interval, with the difference deemed non-significant if the points are within it.

#### Stability of the network across time

To compare network stability across time points, the *NetworkComparisonTest* package is employed to specifically estimate their variance in terms of the global network structure, the global strength of the nodes, edges and centrality. Each of these tests is carried out in succession, with the latter two tests only being conducted by the package if the first two detected significant differences (i.e., if the networks across the two time points do not differ significantly, there is no point examining differences in more specificity; [52]). *P*-values less than 0.05 for these tests indicate significant differences.

## Results

### Network generation and stability

**Fig. 1:**
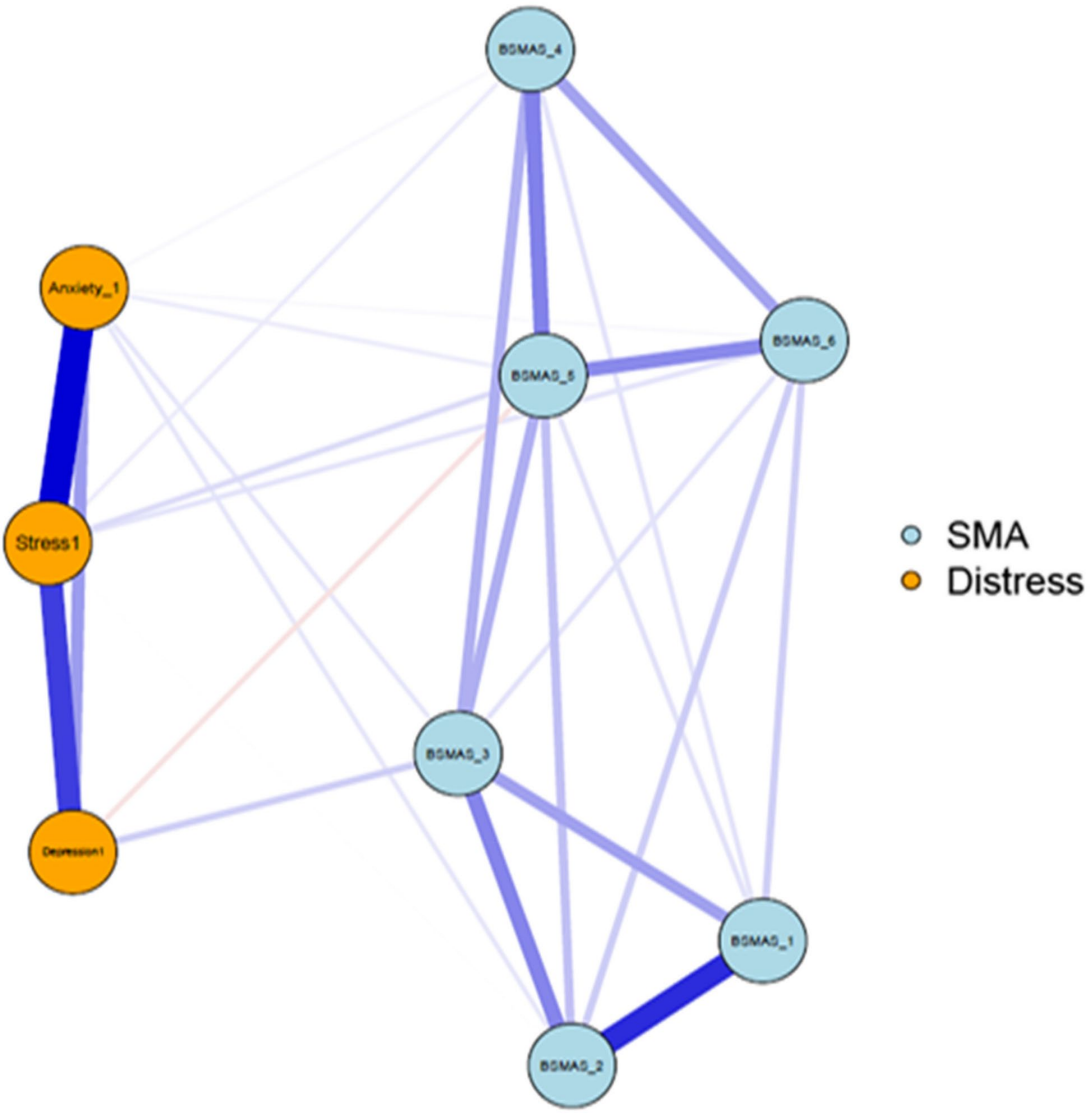
Network of the BSMAS symptoms and DASS subscales at time point 1

**Fig. 2:**
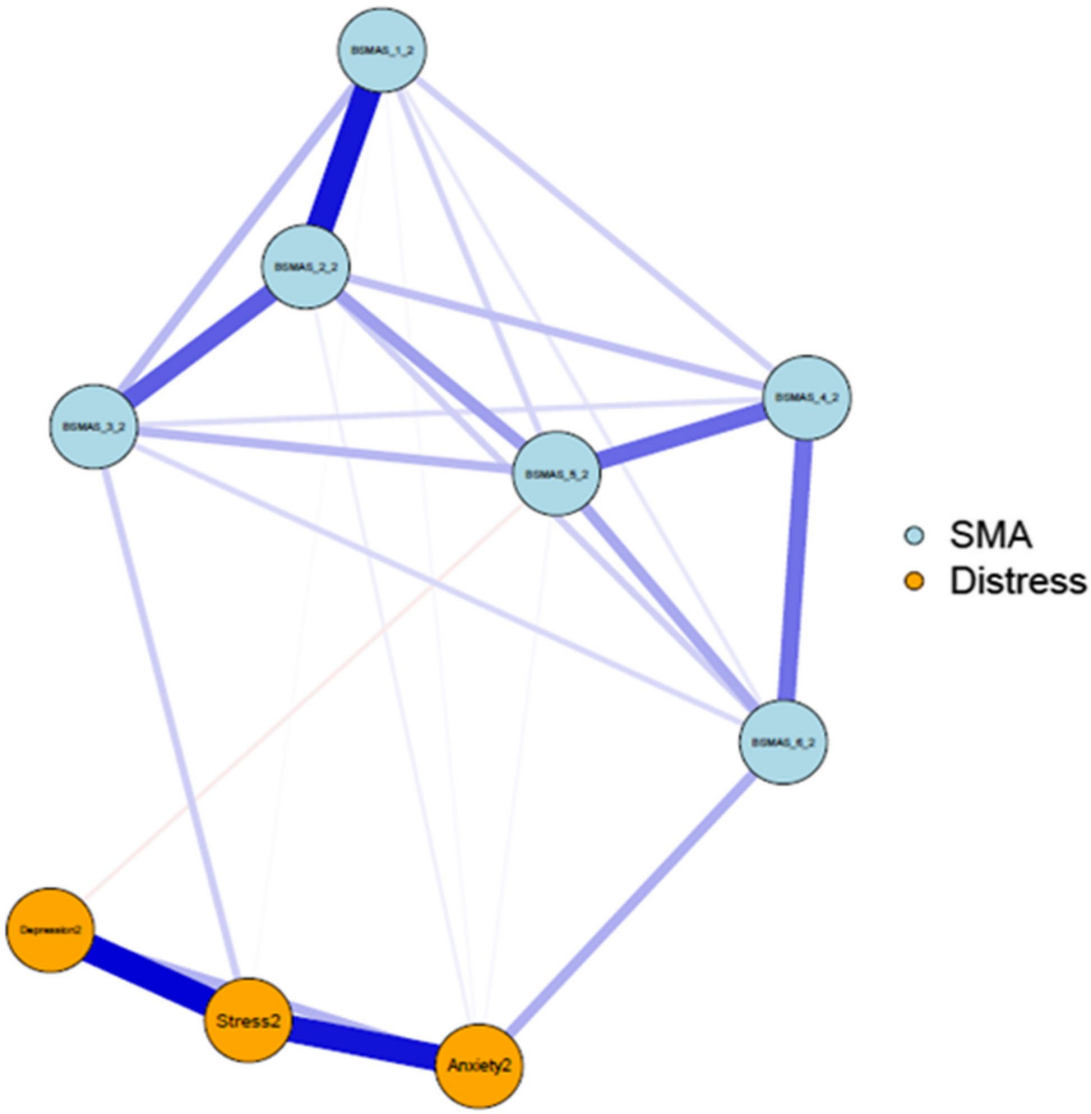
Network of the BSMAS symptoms and DASS subscales at time point 2

Network Analyses generated two networks, one for each timepoint, depicted in Figs. 1 and 2. Edge strengths and calculated centrality statistics for time point 1 are featured in Tables 2 and 3, and for time point 2 in Tables 4 and 5. Note that within the following figures, the BSMAS symptoms of salience, tolerance, mood modification, relapse, withdrawal and conflict are referred to as BSMAS_1, BSMAS_2, BSMAS_3, BSMAS_4, BSMAS_5 and BSMAS_6 respectively.

**Table 2:** Edge strengths across the network of time point 1

	Salience	Tolerance	Mood-Modification	Relapse	Withdrawal	Conflict	Depression	Anxiety	Stress
Salience	0	0.43	0.19	0.06	0.06	0.09	0	0	0
Tolerance	0.43	0	0.25	0.06	0.13	0.10	0	0.05	0.01
Mood-Modification	0.19	0.25	0	0.17	0.16	0.06	0.09	0.05	0
Relapse	0.05	0.05	0.17	0	0.26	0.19	0	0.02	0.04
Withdrawal	0.05	0.12	0.16	0.26	0	0.24	-0.06	0.04	0.07
Conflict	0.09	0.10	0.05	0.19	0.24	0	0	0.01	0.05
Depression	0	0	0.09	0	-0.06	0	0	0.20	0.40
Anxiety	0	0.04	0.04	0.02	0.04	0.01	0.20	0	0.53
Stress	0	0.01	0	0.03	0.07	0.05	0.40	0.53	0

**Table 3:** Centrality statistics across all nodes at time point 1

	node	measure	value
1	Salience	Betweenness	-0.80
2	Tolerance	Betweenness	-0.55
3	Mood-Modification	Betweenness	1.93
4	Relapse	Betweenness	-0.80
5	Withdrawal	Betweenness	0.19
6	Conflict	Betweenness	-0.80
7	Depression1	Betweenness	0.69
8	Anxiety1	Betweenness	-0.80
9	Stress1	Betweenness	0.93
10	Salience	Closeness	-0.12
11	Tolerance	Closeness	0.52
12	Mood-Modification	Closeness	1.93
13	Relapse	Closeness	-0.02
14	Withdrawal	Closeness	0.94
15	Conflict	Closeness	-0.51
16	Depression1	Closeness	-0.61
17	Anxiety1	Closeness	-1.38
18	Stress1	Closeness	-0.74
19	Salience	Strength	-0.49
20	Tolerance	Strength	0.95
21	Mood-Modification	Strength	0.55
22	Relapse	Strength	-0.96
23	Withdrawal	Strength	0.88
24	Conflict	Strength	-1.181
25	Depression1	Strength	-1.14
26	Anxiety1	Strength	-0.08
27	Stress1	Strength	1.46
28	Salience	Expected Influence	-0.26
29	Tolerance	Expected Influence	1.04
30	Mood-Modification	Expected Influence	0.68
31	Relapse	Expected Influence	-0.69
32	Withdrawal	Expected Influence	0.16
33	Conflict	Expected Influence	-0.89
34	Depression1	Expected Influence	-1.68
35	Anxiety1	Expected Influence	0.11
36	Stress1	Expected Influence	1.51

**Table 4:** Edge strengths across the network of time point 2

	Salience	Tolerance	Mood-Modification	Relapse	Withdrawal	Conflict	Depression	Anxiety	Stress
Salience	0	0.45	0.14	0.09	0.08	0.04	0	0.01	0.01
Tolerance	0	0.45	0.14	0.09	0.08	0.04	0	0.01	0.01
Mood-Modification	0.45	0	0.31	0.12	0.18	0.09	0	0.03	0
Relapse	0.14	0.31	0	0.07	0.14	0.07	0	0	0.09
Withdrawal	0.09	0.12	0.07	0	0.29	0.27	0	0	0
Conflict	0.08	0.18	0.14	0.29	0	0.16	-0.03	0.02	0
Depression	0.04	0.09	0.07	0.27	0.16	0	0	0.16	0
Anxiety	0	0	0	0	-0.03	0	0	0.15	0.49
Stress	0.01	0.03	0	0	0.02	0.16	0.15	0	0.46

**Table 5:** Centrality statistics across all nodes at time point 2

	node	measure	value
1	Salience	Betweenness	-1.11
2	Tolerance	Betweenness	0.78
3	Mood-Modification	Betweenness	0.51
4	Relapse	Betweenness	-1.11
5	Withdrawal	Betweenness	-0.57
6	Conflict	Betweenness	0.51
7	Depression	Betweenness	-1.11
8	Anxiety	Betweenness	0.51
9	Stress	Betweenness	1.59
10	Salience	Closeness	-1.39
11	Tolerance	Closeness	0.51
12	Mood-Modification	Closeness	0.46
13	Relapse	Closeness	0.92
14	Withdrawal	Closeness	0.80
15	Conflict	Closeness	0.97
16	Depression	Closeness	-1.72
17	Anxiety	Closeness	-0.41
18	Stress	Closeness	-0.13
19	Salience	Strength	-0.39
20	Tolerance	Strength	2.02
21	Mood-Modification	Strength	-0.40
22	Relapse	Strength	-0.24
23	Withdrawal	Strength	0.15
24	Conflict	Strength	-0.52
25	Depression	Strength	-1.34
26	Anxiety	Strength	-0.41
27	Stress	Strength	1.13
28	Salience	Expected Influence	-0.26
29	Tolerance	Expected Influence	1.96
30	Mood-Modification	Expected Influence	-0.28
31	Relapse	Expected Influence	-0.13
32	Withdrawal	Expected Influence	-0.18
33	Conflict	Expected Influence	-0.39
34	Depression	Expected Influence	-1.56
35	Anxiety	Expected Influence	-0.29
36	Stress	Expected Influence	1.14

The network at time point one showed excellent stability in terms of its basic structure (edge stability coefficient = 0.75, expected influence centrality stability coefficient = 0.60) and marginal stability regarding secondary measures of centrality (closeness centrality stability coefficient = 0.13, betweenness centrality stability coefficient = 0.05). In terms of bridges between network clusters, stability ranged from acceptable (bridge expected influence stability coefficient = 0.36), to marginal (bridge betweenness stability coefficient = 0.0) to insufficient (bridge closeness stability coefficient = 0.0).

These structural network characteristics were shared with the network at time point two both in terms of basic structure (edge stability coefficient = 0.75, expected influence centrality stability coefficient = 0.60) and secondary measures of centrality (closeness centrality stability coefficient = 0.13, betweenness centrality stability coefficient = 0.05). Though the bridges between clusters featured greater stability than time point 1 (bridge expected influence stability coefficient = 0.52, bridge betweenness = 0.05, bridge closeness = 0.21).

With all necessary structural measure’s stability within acceptable limits, further analysis of the network structures and network comparison was undertaken. However, given the marginal to unacceptable stability of both closeness and betweenness as measures of centrality, it was deemed that results from these measures cannot be safely generalised, or safely used to draw inferences about the data. Thus, these measures are only considered in the following as potential indicators that may point to avenues of further investigation, unless a result of 0.0 was scored on their stability coefficient, in which case they are completely disregarded.

### Network characteristics at Time Point 1

**Fig. 3:**
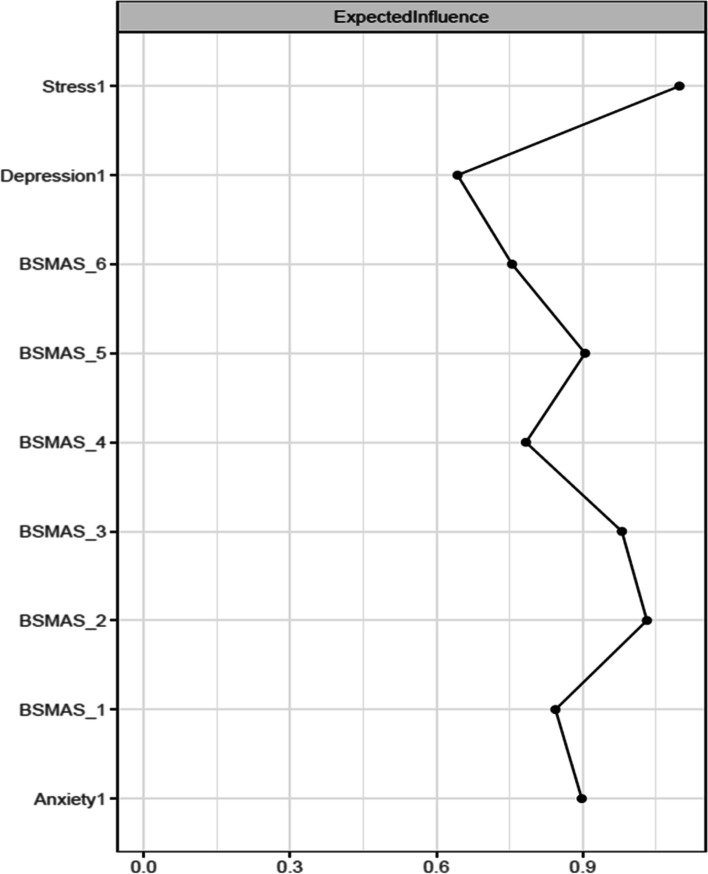
Expected Influence across all nodes at time point 1

**Fig. 4:**
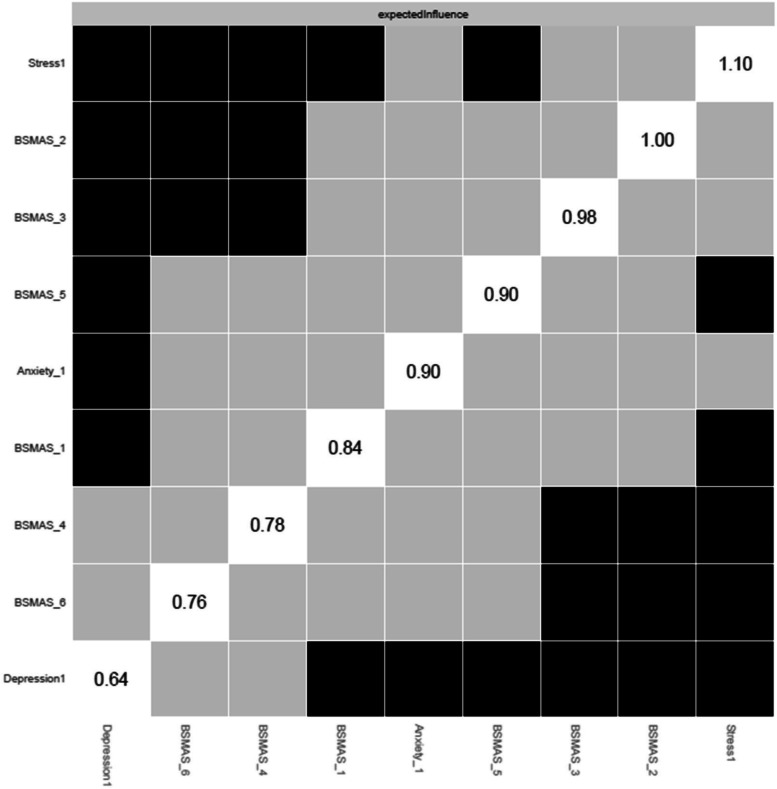
Centrality difference tests of Expected Influence at time point 1

Figure 3 depicts the expected influence of all nodes at time point 1, and Fig. 4 depicts centrality difference tests determining the significance of differences in expected influence between all nodes, with black squares indicating significant differences. In terms of overall centrality, stress had the most and strongest connections with other nodes. Stress had expected influence significantly greater than the majority of nodes, with the exception of anxiety and the BSMAS symptoms of tolerance and mood modification (Items 2 & 3). These BSMAS symptoms formed a consistent plateau of centrality, significantly above the symptoms of Relapse and Withdrawal (Item 4 & 5 respectively). Depression was relatively low in centrality, with a result significantly lower than every other node except relapse and withdrawal.

**Fig. 5:**
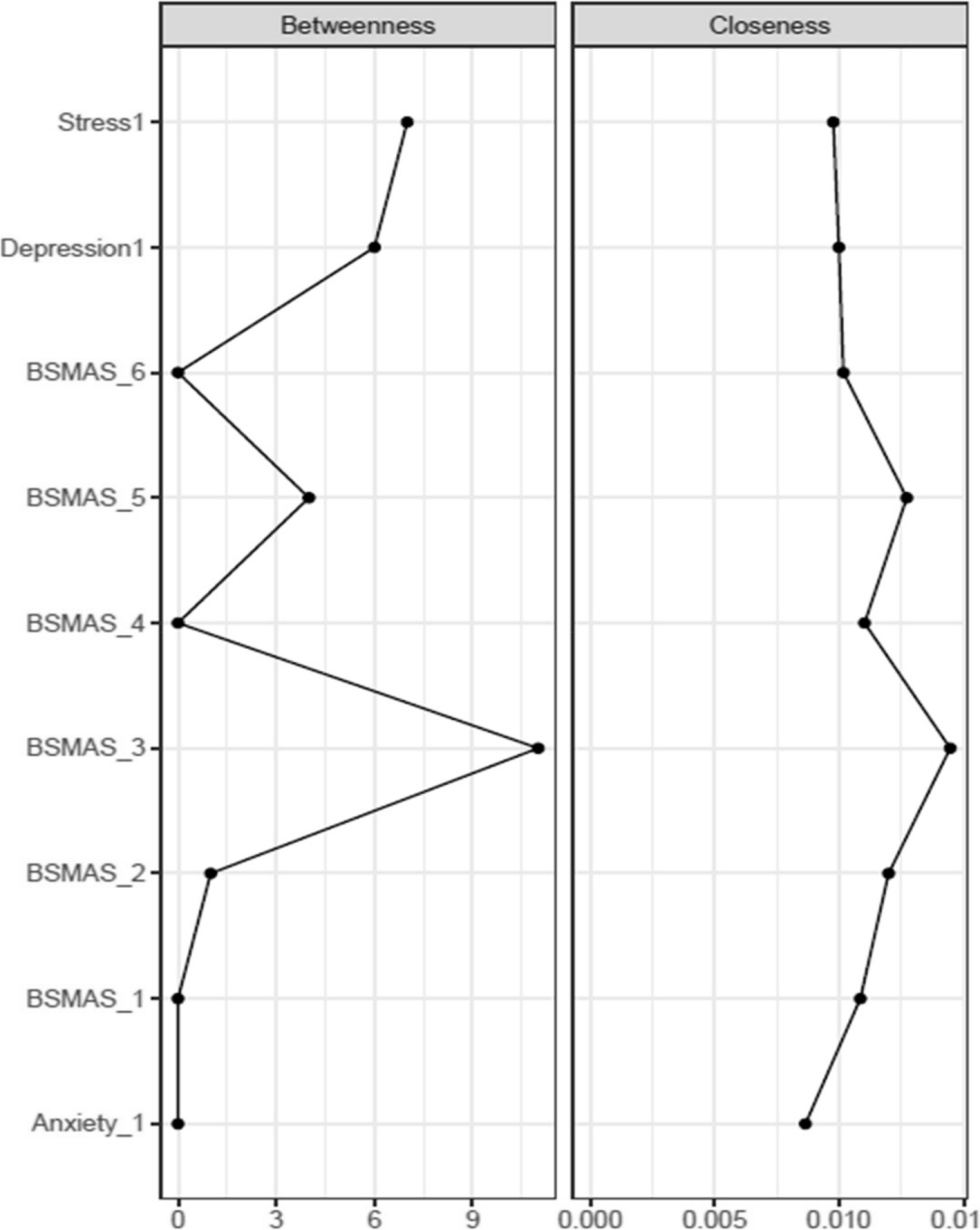
Closeness and betweenness across all nodes at time point 1

**Fig. 6:**
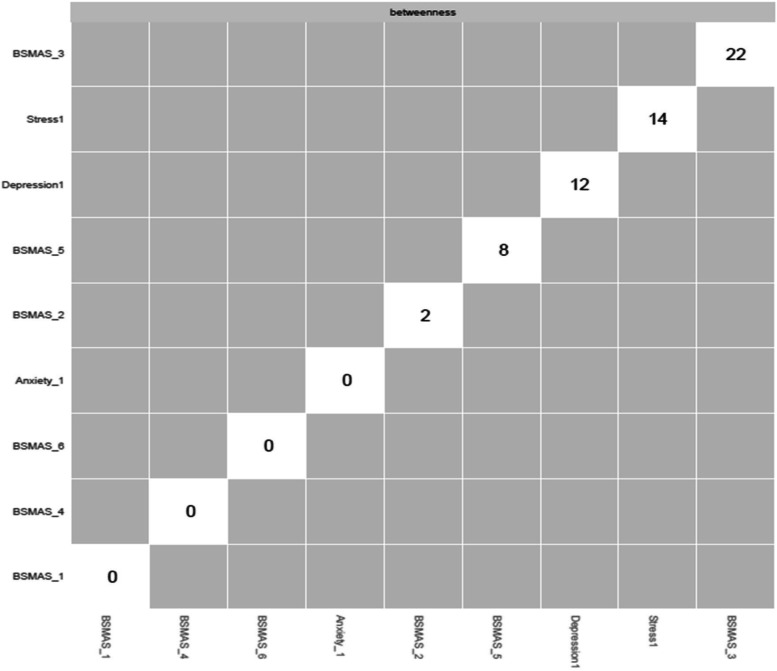
Centrality difference tests of betweenness at time point 1

**Fig. 7:**
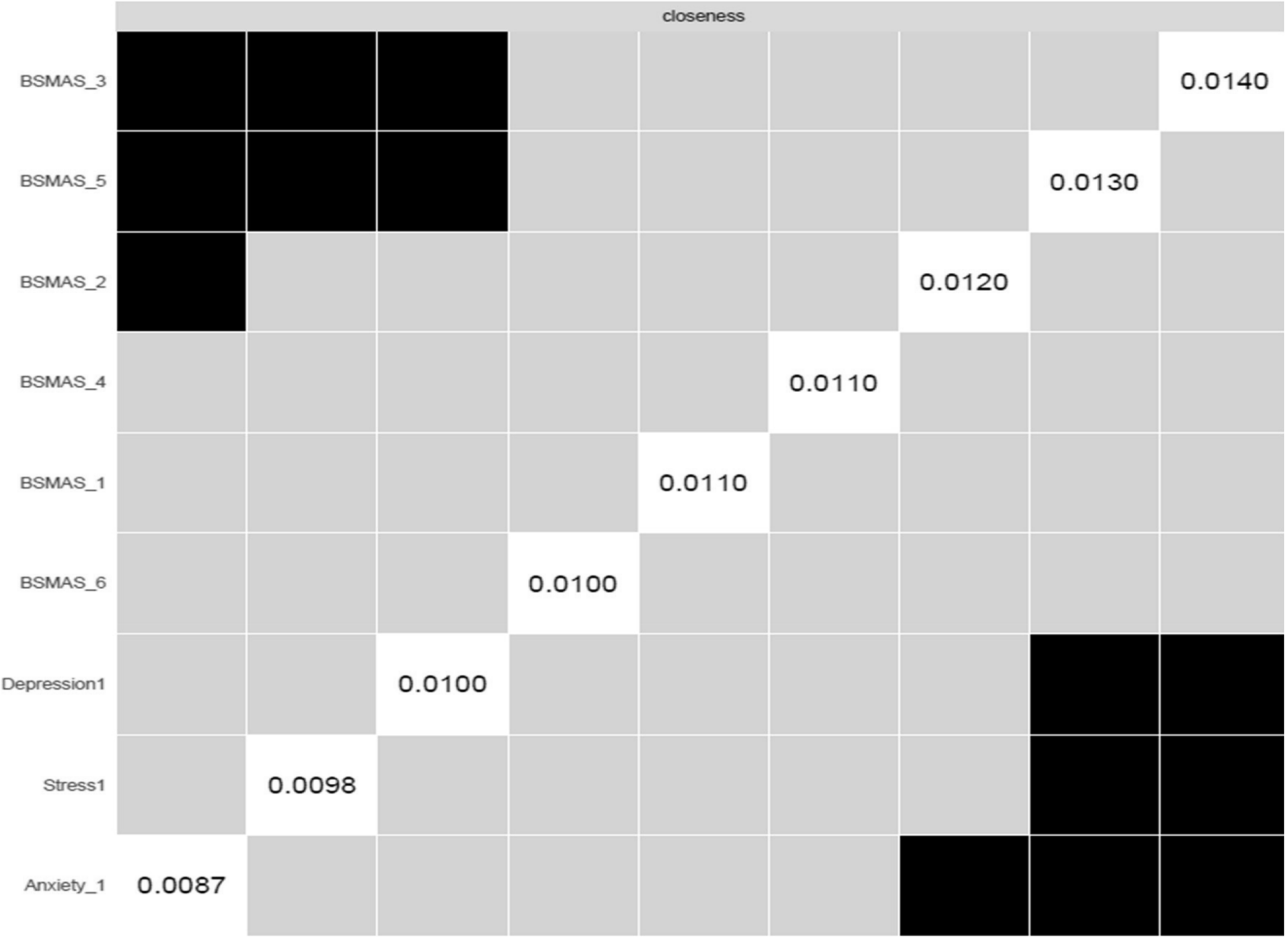
Centrality difference tests of closeness at time point 1

Accordingly, Fig. 5 depicts nodes’ closeness and betweenness at time point 1, while Figs. 6, 7 depict centrality difference tests determining the significance of differences in betweenness and closeness, with black squares indicating a significant difference. In terms of the number of times a node was on the shortest path (i.e., betweenness), there were no significant differences. In terms of the distance between nodes (i.e., closeness), BSMAS symptoms of mood modification and withdrawal displayed the greatest centrality, with each displaying significantly higher centrality in the network than the DASS subscales.

**Fig. 8:**
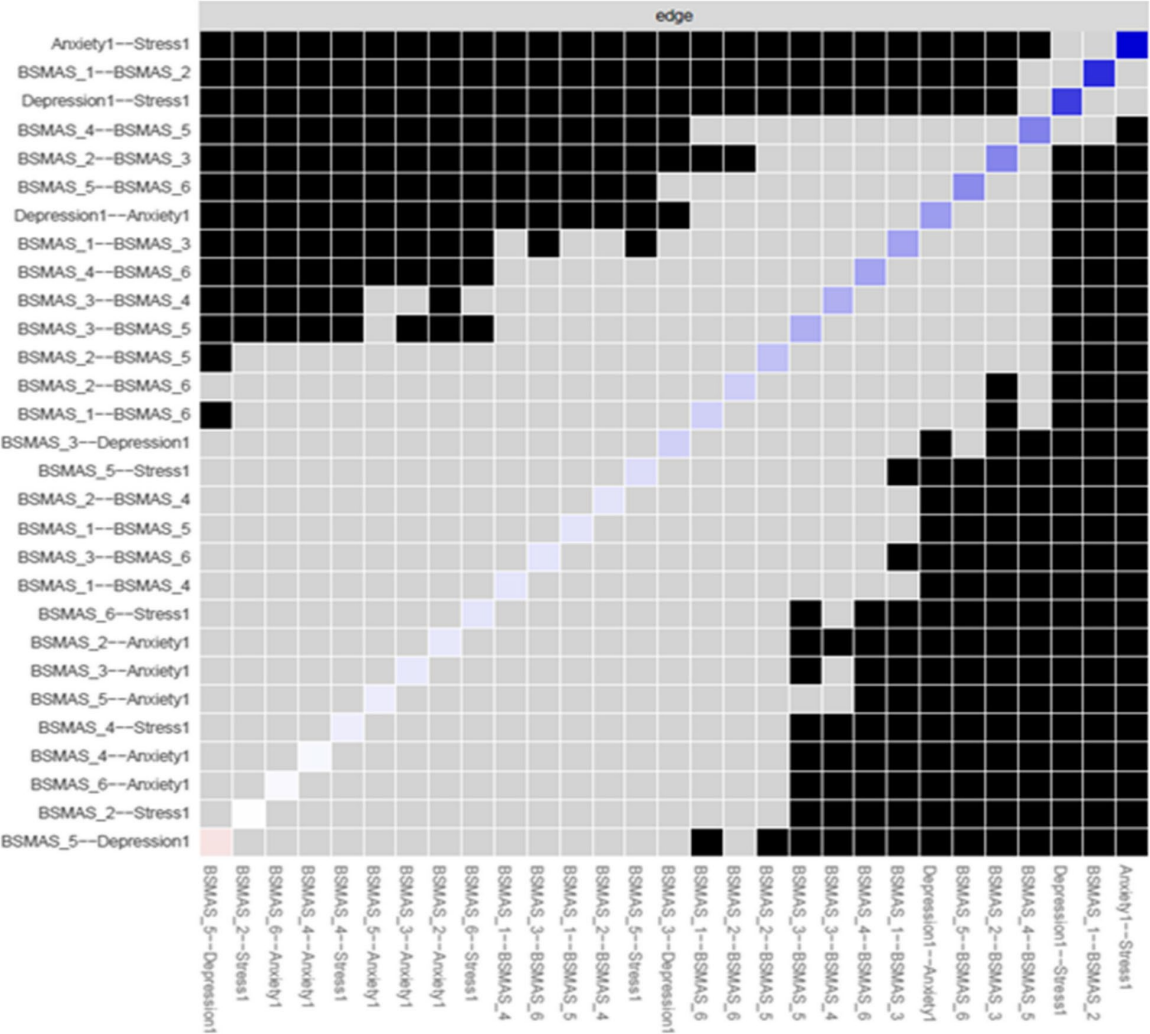
Edges’ difference tests at time point 1

Figure 8 depicts edge difference tests, indicating that the edges between anxiety and stress, depression and stress, and between the BSMAS symptoms of salience and tolerance were significantly stronger than those of other nodes.

### Bridge characteristics at Time Point 1

**Fig. 9:**
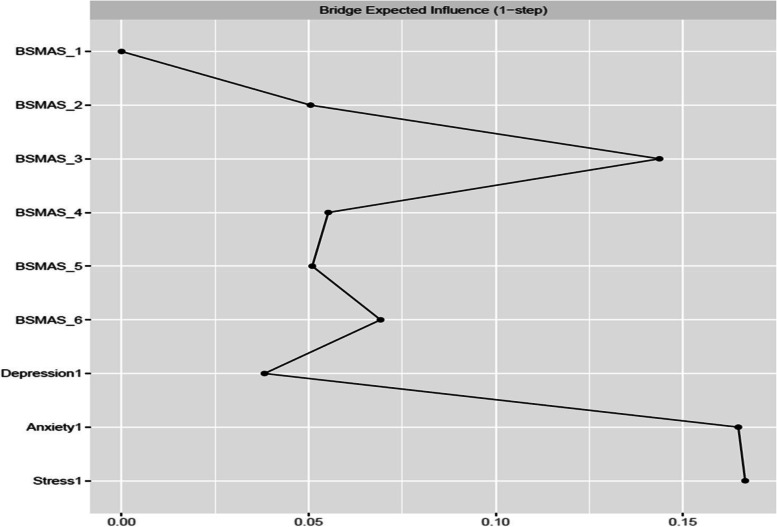
Bridge Expected Influence Centrality at time point 1

**Fig. 10:**
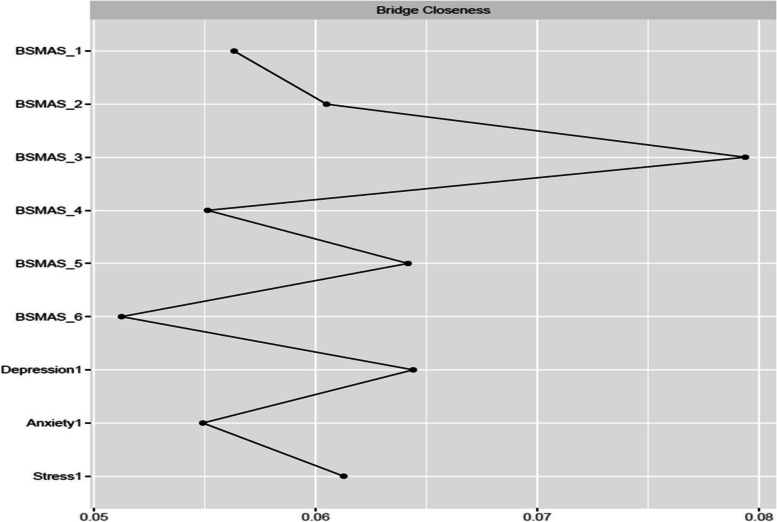
Bridge Closeness Centrality at time point 1

Figures 9 and 10 depict bridge expected influence, closeness and betweenness centralities between the BSMAS symptoms and the DASS subscales. SMA symptoms of mood modification and conflict demonstrated markedly higher expected influence connections with the DASS subscales cluster than other SMA symptoms. With regards to the DASS subscales, anxiety and stress were in a similar position, with a bridge expected influence on the BSMAS symptoms substantially greater than that of depression (see Fig. 9). In terms of the proximity/closeness between nodes in the two subgroups, the BSMAS symptom of mood modification (Item 3) and withdrawal (Item 5) were the most proximal to the distress subgroup, with depression serving as the closest connecting point.

### Network characteristics at Time Point 2

**Fig. 11:**
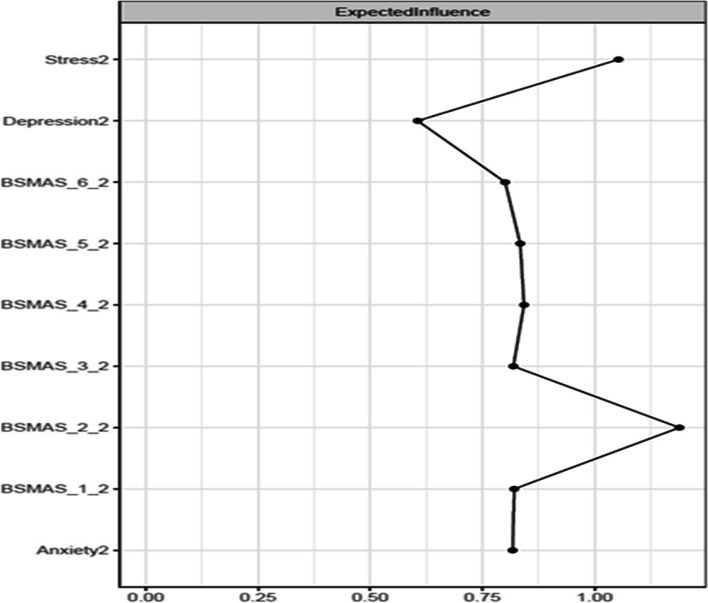
Expected Influence across all nodes at time point 2

**Fig. 12:**
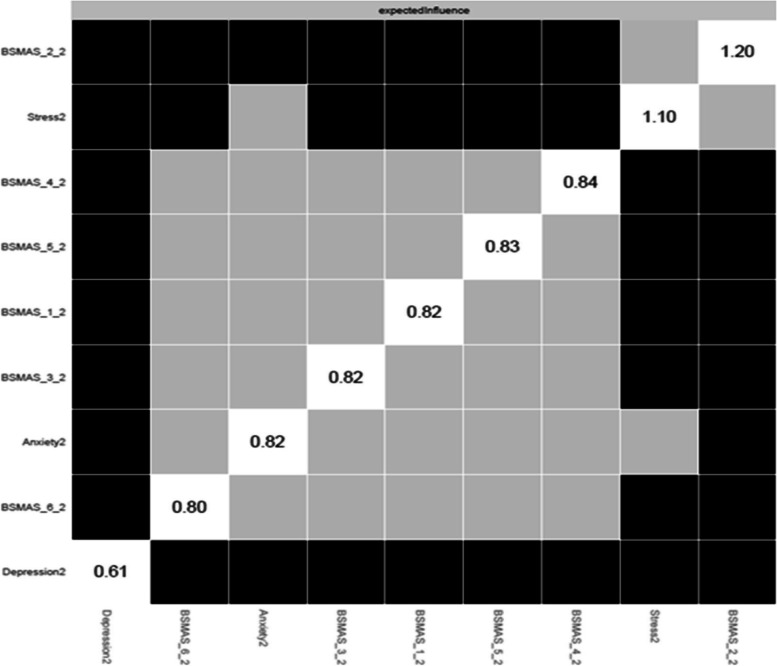
Centrality difference tests of Expected Influence at time point 2

Figure 11 depicts the expected influence of all nodes at time point 2, whilst Fig. 12 depicts the significance of nodes’ differences in terms of their expected influence. The highest overall centrality in terms of expected influence was demonstrated by the BSMAS symptom of tolerance (Item 2), which was closely followed by the DASS subscale of stress. As is evidenced in Fig. 12, both stress and tolerance were significantly greater in their expected influence centrality than the other network nodes.

**Fig. 13:**
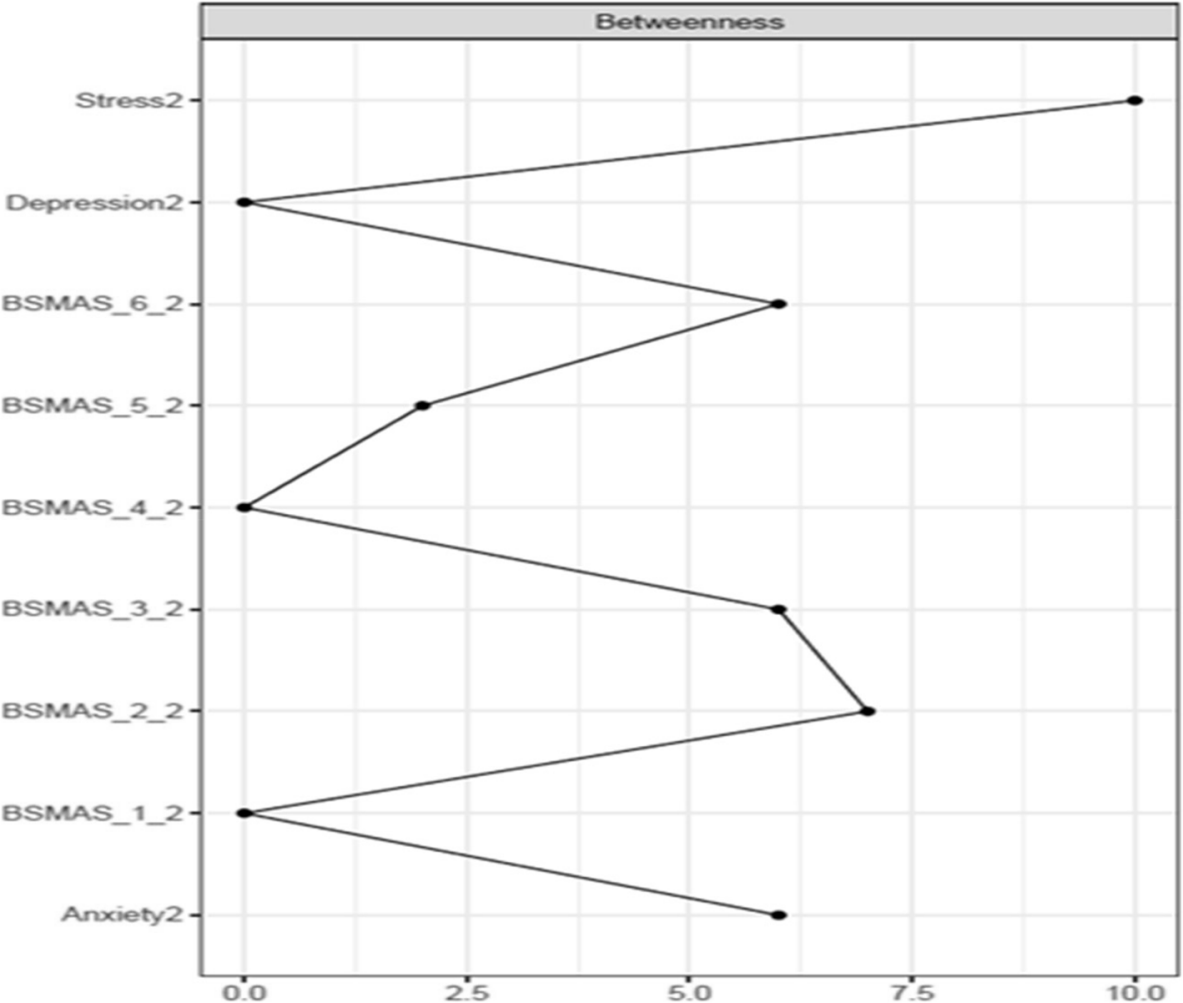
Betweenness across all nodes at time point 2

**Fig. 14:**
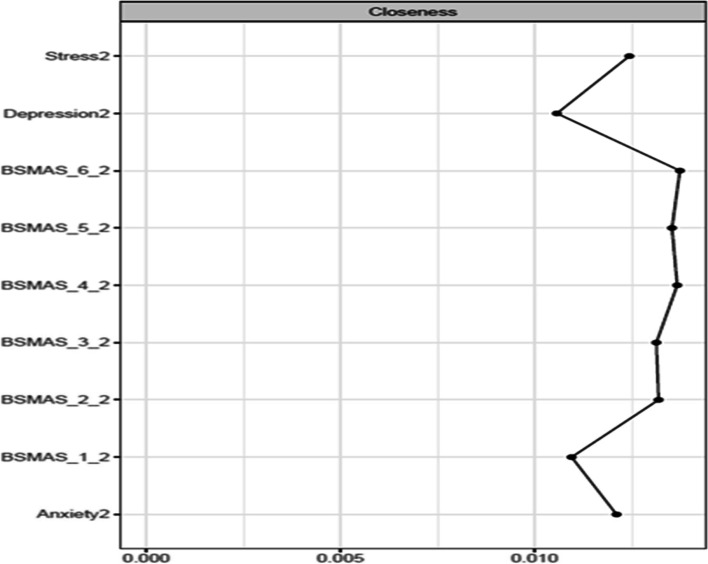
Closeness across all nodes at time point 2

**Fig. 15:**
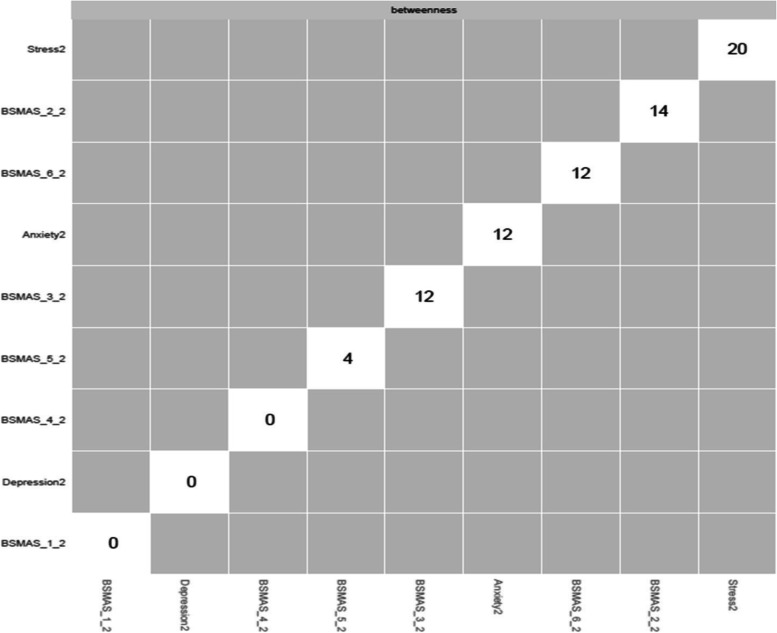
Centrality difference tests of betweenness at time point 2

**Fig. 16:**
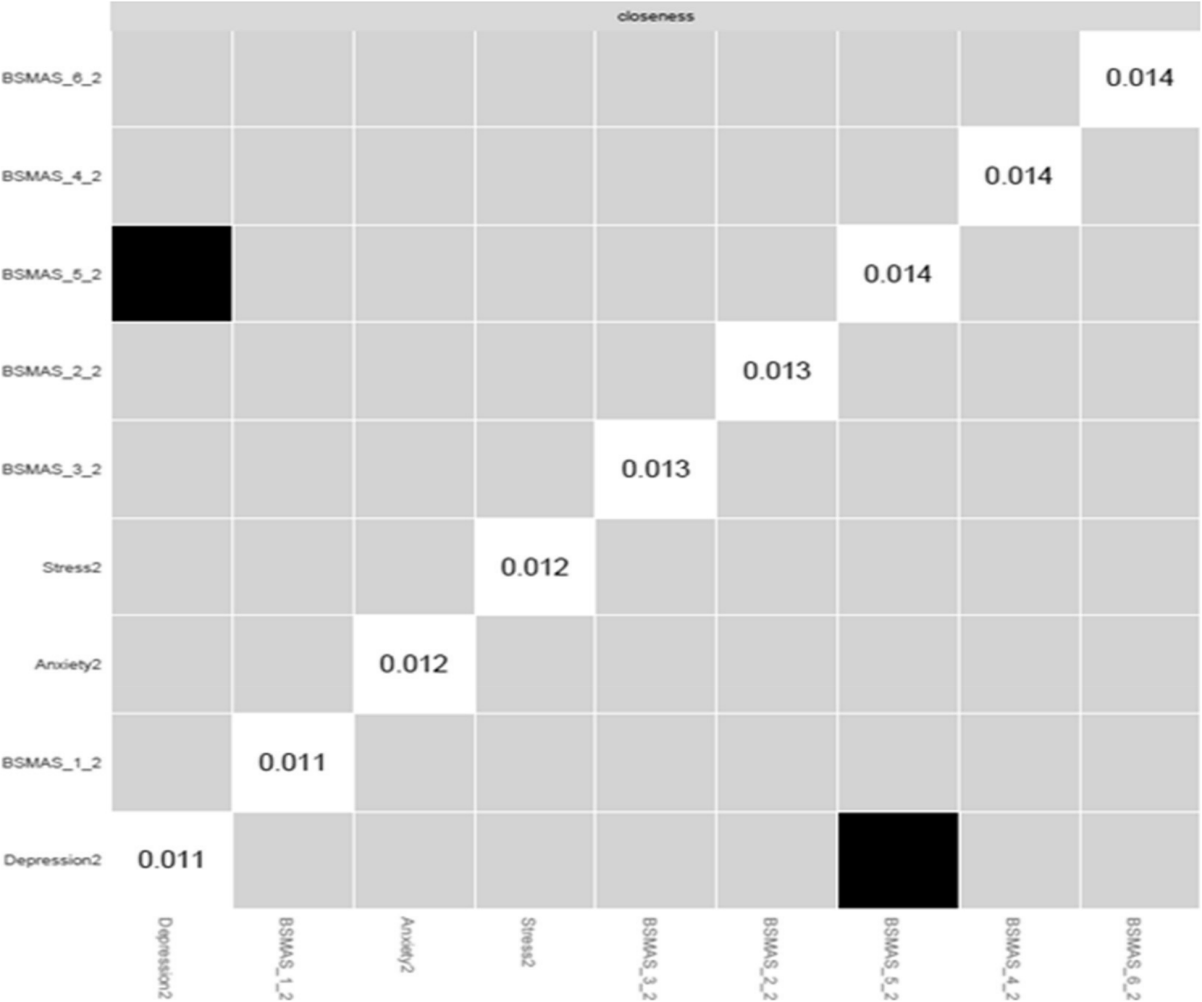
Centrality difference tests of closeness at time point 2

Figures 13 and 14 depict the betweenness and closeness respectively of all nodes at time point 2, whilst Figs. 15 and 16 depict centrality difference tests determining the significance of differences in betweenness and closeness respectively. No significant differences in the number of times a node was on the shortest path (i.e., betweenness) identified between the nodes, nor were there any nodes significantly higher in closeness, with the exception of withdrawal (Item 5).

**Fig. 17:**
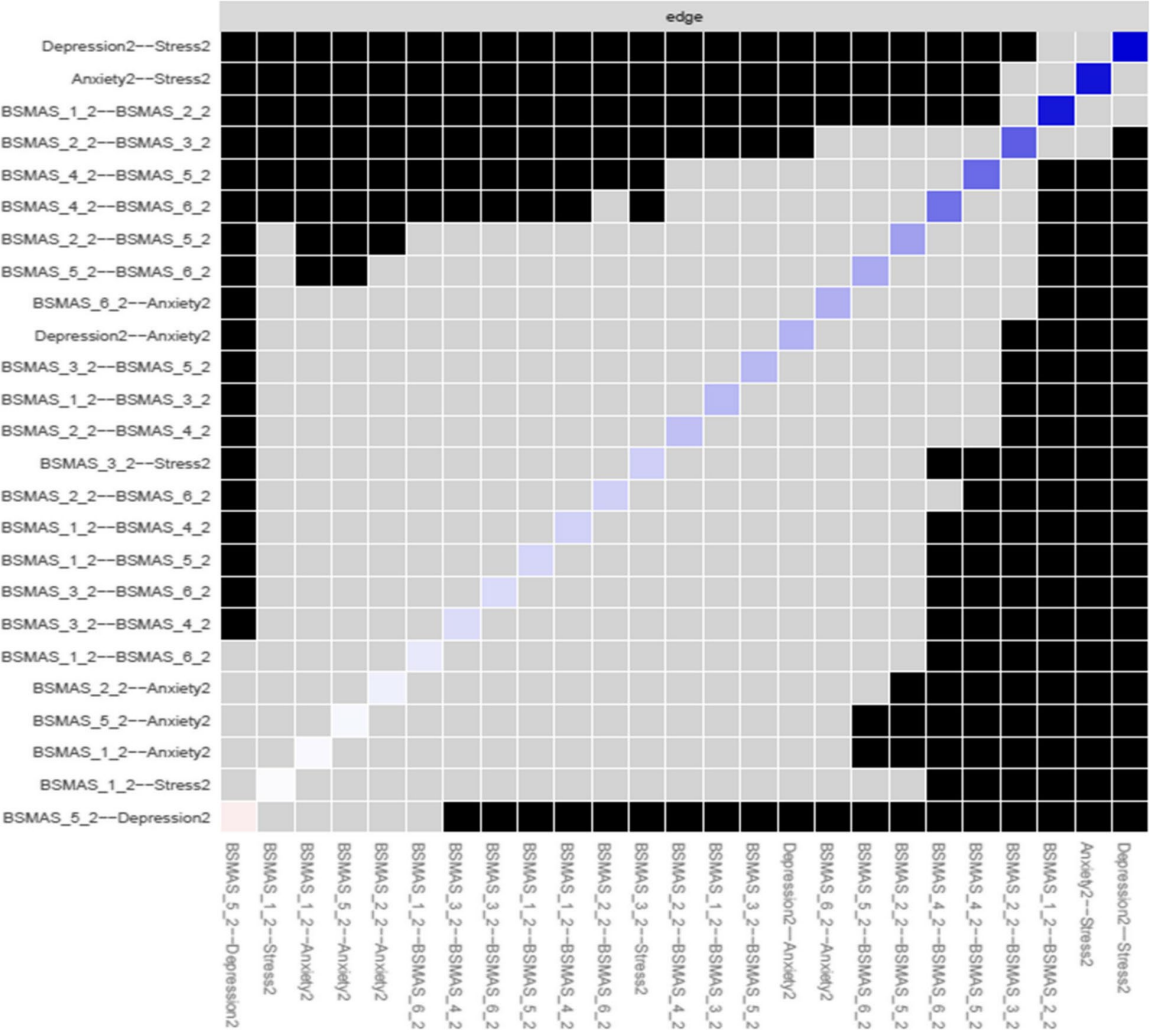
Edges’ difference tests at time point 2

Figure 17 depicts edge difference tests at time point 2. As with time point 1, the edges between anxiety and stress, depression and stress, and between the BSMAS symptoms of salience and tolerance (Items 1 & 2) were significantly stronger than those between other nodes. Additionally, the connection between the BSMAS symptoms of tolerance and mood modification (Items 2 & 3) was a significantly stronger connection than over half of those assessed.

### Bridge characteristics at Time Point 2

**Fig. 18:**
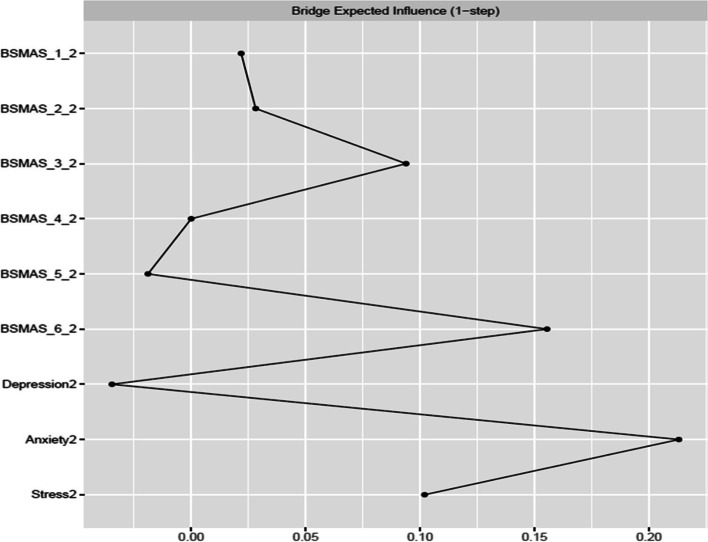
Bridge Expected Influence Centrality at time point 2

**Fig. 19:**
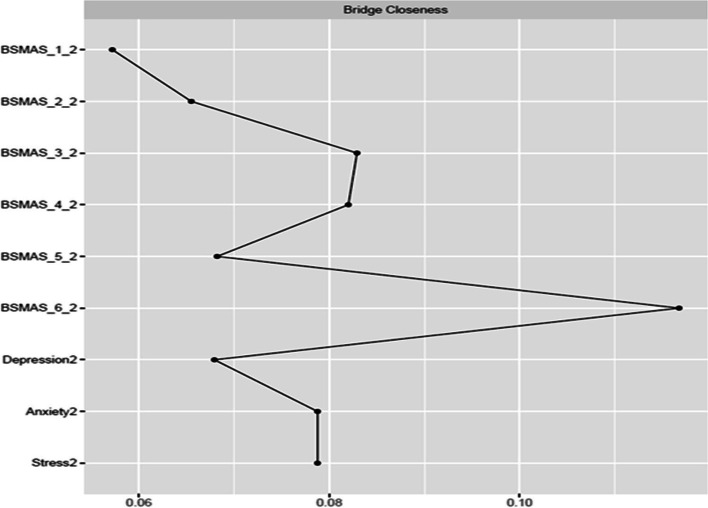
Bridge Closeness Centrality at time point 2

**Fig. 20:**
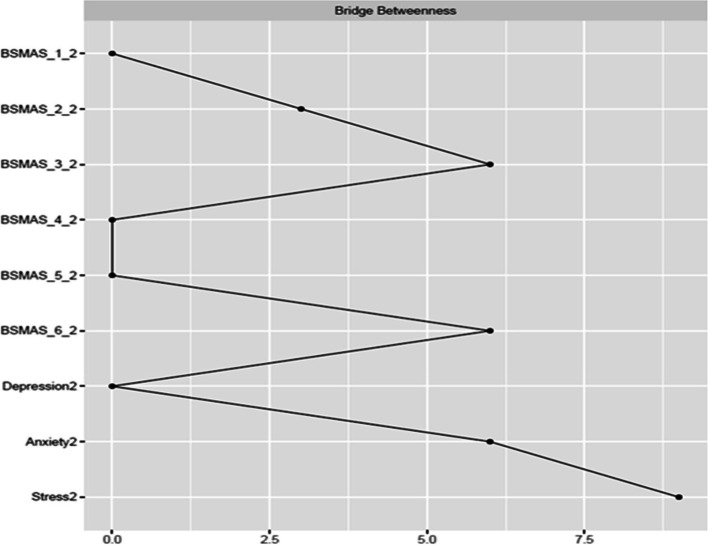
Bridge Betweenness Centrality at time point 2

Figures 18, 19 and 20 depict bridge centralities between the BSMAS symptoms cluster and the DASS subscales cluster at time point 2. As in time point 1, the SMA symptoms of mood modification (Item 3) and conflict (Item 6) bridged the SMA behaviours cluster to the DASS subscales cluster via the nodes of anxiety and stress. These results were displayed in both the number and strength of connections between these nodes (expected influence centrality) and the number of times these nodes were used as connecting joints in paths between other nodes in these two networks (betweenness centrality). Further, in terms of the proximal distance between nodes in the two subgroups, the BSMAS symptom of conflict was the most central symptom, with anxiety and stress being the most proximal distress experiences.

### Longitudinal network comparison

Finally, a network invariance test revealed no significant differences between the network at time point 1 and time point 2 in terms of global network invariance (*p* = 0.36) and global strength Invariance (*p* = 0.42).

## Discussion

The rapid expansion of social media use has generated concerns regarding the development of PSMU behaviours. These have been noted to closely resemble those displayed in substance/behavioural addictions [1, 2]. In that line, a portion of scholars have defined these behaviour as social media addiction (SMA) and have advocated in favour of describing it via the lenses of the components model of addiction framework (i.e. salience; mood-modification; tolerance; relapse; withdrawal; losing of interest into other activities/functional impairment; [1, 9]. Such suggestions have been criticised as accommodating the risk of pathologizing common everyday behaviours, such as the use of social media, and lacking validity due to adhering to substance abuse criteria/behaviours that may fail to correctly depict this emerging condition [19, 20]. Additionally, there is a lack of clarity regarding the details of links between excessive use symptoms and markers of impairment, such as distress, which cause further doubts [19, 20]. Finally, the occurrence of SMA behaviour as an independent diagnostic condition has been contested on the basis of SMA related behaviours constituting biproducts/ secondary symptoms of primarily distress conditions such as depression, anxiety and stress [19, 20].

To address these concerns, the current research innovated via longitudinally assessing a normative cohort of adult social media users twice over a period of two years considering concurrently their SMA and depression, anxiety and stress self-reported experiences. Advanced longitudinal network analysis models, enriched via the LASSO algorithm, were calculated for both time points [29, 32]. These aimed to firstly clarify whether SMA criteria, as described on the basis of the components model of addiction, formed indeed an underpinning network of behaviours, stable over time and across different sample compositions [10]. Answering this question would indicate that the construct is rather formative and not reflective (i.e., it is not just a conception of scholars or a sample specific construct, while it is steadily reflected the same way over time [19, 20]).

Secondly, the analysis aimed to dispel to what extent SMA behaviours may mix/blend or closely relate to distress behaviours such as depression, anxiety and stress [53]. If the latter was to be true, then the SMA and distress components of the network would be expected to mix and not to represent distinctly different network clusters (i.e. SMA and distress related behaviours would represent different behavioural network clusters and thus should be classified independently). Thirdly, it was aimed to identify key/central/pivotal behaviours in the broader network, that should be prioritized in prevention and/or intervention for those presenting with SMA and/or comorbid depression, anxiety and stress (i.e. central nodes of the network with higher expected influence). Findings indicated that SMA behaviours/criteria, as per the components model of addiction, do constitute a formative network of symptoms, which is not sample or time specific. Furthermore, the SMA behaviours cluster was distinct to that of depression, anxiety and stress experiences across both measurements, favouring its classification as an independent diagnostic condition. Lastly, mood modification appeared to be consistently (across both time points) a central network node and has been facilitating as the main bridge primarily with distress symptoms of stress and anxiety rather than depression.

### SMA and distress network

As summarized prior, results portrayed a stable overtime network cluster of SMA symptoms, which is associated yet distinct, to the distress related cluster of nodes composed by depression, anxiety and stress. These findings appear to align with the recent SMA, cross-sectional, network analysis study of Romanian data, which also supported the SMA defined behaviours of salience, tolerance, mood-modification, withdrawal, relapse and functional impairment being closely related and informing a clear cluster of nodes [35]. Therefore, the present study argues in favour of the idea of SMA operating as a formative construct, which occurs independently of the conception of scholars (i.e. does not only reflect theoretical conceptualizations [19, 20]. This provides an indication in favour of those who support the SMA conceptualization and potentially the introduction of a distinct diagnostic category to capture the syndrome [35, 36]. In that context, SMA behaviours related to mood-modification appeared to be central across both time points, reinforcing the idea of addictions, such as SMA, acting the problematic solution (e.g., way to either experience more positive or buffer negative emotions) of the distress generated by other problems [53]. Nevertheless, one cannot exclude the need of additional nodes, such as those likely reflecting “deception behaviours associated to the use of social media” (e.g. an individual concealing the amount of time they consume on social media usage) and/or relationship difficulties (e.g. as with other forms of addictions, a person may be marginalized within their social surrounding) to better describe the phenomenon [54]. Thus, although findings support the six, adjusted to the abuse of social media, addiction criteria operating as a distinct, SMA underpinning, formative network, the need for additional behavioural nodes to better describe the condition cannot be excluded.

Despite these, and in contrast to the results of the Stănculescu [35] Romanian study, where salience and withdrawal were identified as the most ‘central’ symptoms, the current study identified tolerance and mood-modification as the most highly central in terms of expected influence and closeness respectively. A possible explanation for this discrepancy may refer to the more rigorous methodology and wider aims applied in the current study, compared to that conducted by Stănculescu [35]. Firstly, the current analysis examined network stability across different resamples (i.e., potential population compositions) and over time (i.e. longitudinally), which was not the case in the Stănculescu [35] study. Secondly, the present study thoroughly examined centrality differences based on t-test comparisons in conjunction with the visual graph/network inspection, whilst such comparisons were not reported in the Romanian study [35]. Thirdly, centrality indices informing the present findings were referring to the extended network of SMA and distress behaviours, and not the narrower network of SMA behaviours only [35]. Thus, it is likely that whilst salience and withdrawal may be more central in the context of SMA behaviours, without taking into consideration concurrent depression, anxiety and stress behaviours; tolerance and mood modification maybe more pivotal in the broader context of SMA and distress comorbidities together. Finally, it is also likely that cultural differences between the two samples may alternate the experience of SMA between the populations, such that withdrawal and salience maybe more central for the Romanian sample [35]. Such differences inevitably invite further investigation regarding the cross-cultural invariance of the SMA network, as with other behavioural addictions related to the abuse of digital media (see gaming disorder [53, 54]).

The current findings were also revealing considering the differential diagnosis concerns referring to SMA behaviours constituting primarily a secondary symptom of distress behaviours related to depression, anxiety and stress, rather than a distinct condition itself [54]. Specifically, network models across both time points consistently revealed two distinguishable clusters of nodes within the broader network, clearly dividing SMA and distress behaviours. Thus, although distress and SMA behaviours appeared related, they were not blended/mixed in a way that would advocate a common classification [41].

Furthermore, the current study also expands available knowledge regarding the relationship between SMA and distress, via the examination of the ‘bridging centrality’ of the various symptoms [54]. Primarily, the connections between the SMA behaviours of mood-modification and conflict, with anxiety and stress, appear to have acted as comorbidity bridges, featuring the highest expected influence bridge centrality values amongst their respective subnetworks (i.e., the number and strength of connections to other subnetworks). In addition, withdrawal symptoms served as a “go-between” in this link between subnetworks, with the highest betweenness bridge centrality (the amount of and strength of the connections between SMA and distress that used it as a go-between). Thus, these findings imply that the need to moderate one’s negative feelings via SMA, and/or the stress/anxiety related to the occurrence of functional impairments in a person’s life (e.g., conflicts with others due to SMA behaviours) could operate as the main connection points in the cyclical relationship between distress and SMA. This hypothesized process aligns with evidence relevant to other behavioural addictions [55]. Thus, one could support that stressed and anxious individuals may excessively use social media to cope with, and to modify their anxious manifestations, suffering conflicts with their real-world obligations and desires as a result of that use. The latter might induce more stress and anxiety, and perhaps even more when withdrawals ensue after failed attempts to reduce use. Further SMA and depression symptoms could follow as a result of the development of conflict/mood-modification and stress/anxiety respectively. This interpretation is reinforced by prior cross-sectional and longitudinal research in the field of addiction psychology that: a) portrays stress, as well as unhealthy coping mechanisms in response to stress, to operate as primary causes of addictions [56–59] and; b) proposes the need to escape from negative moods as highly associated to addictive tendencies [6]. These results may thus imply, that clinicians treating clients with comorbid SMA/distress, may wish to target these bridging symptoms in particular, in order to cut any possible bidirectional feedback loops between these disorders.

On a separate note, the depression node was found to display a seeming lack of importance in the network. Specifically, depressive behaviours were shown to possess significantly lower general centrality and bridge centrality, implying that they may not have as a formative effect on the experience of SMA symptoms, as stress and anxiety. Furthermore, depression displayed a negative association with withdrawal symptoms, the only negative association in the network. While initially this may seem to contradict prior research associating depression and social media use [41], this is not necessarily the case. Depression still displayed a positive association with the symptom of mood-modification, accommodating prior research linking addiction with the use of social media as a relief mechanism [6]. Furthermore, while at first it might seem oxymoronic that the experience of depression might associate with a reduction in SMA withdrawal symptoms, this may not be the case. It is likely that, as with other addictions, those experiencing depression are less able to attempt containing their addictive patterns, whilst when/if they do make attempts, those attempts may be less successful and thus they do not experience withdrawal [60]. Those experiencing depression have depressed mood, lack of energy and a lack of motivation all of which negate action and make it harder to quit or make an attempt to cease problematic behaviours [12, 16]. Furthermore, a lack of direct impact of depressive experiences on SMA symptoms in the network does not imply a lack of impact overall. In the current findings, depression still displayed very strong relationships with stress and anxiety, allowing it to influence SMA via its influence on these symptoms. However, as causality associations were not directly explored in the current study, these interpretations require further additional evidence to be better supported.

### Limitations and further study recommendations

Despite the relevant findings reported here, such conclusions and implications may need to be considered in the light of the several limitations of the present study. Firstly, a convenience, community, western/English speaking sample of adult social media users was collected, potentially restricting the generalization of the findings to non-western, children-adolescent and clinical populations. Secondly, findings were exclusively based on self-reported, psychometric scales and thus risks of subjectivity or self-reporting errors cannot be excluded. Therefore, considering that there is evidence of objectively measuring social media use [61, 62] future researchers may wish to consider examining non-adult, non-western and/or clinical samples via multimethod designs entailing additionally physical actigraphy and/or digital monitoring means to further expand the available knowledge. Thirdly, this study focused exclusively on the network between PSMU and distress; however, other variables have been associated with PSMU and should be considered in future studies (e.g., fear of missing out [63]).

## Conclusions and implications

Overall, the findings of the present study appear to have added important knowledge across three areas surrounding problematic social media usage. These involve the conceptualization of this debated condition, its differential diagnosis and key behavioural symptoms informing it [34, 48]. In particular, the current findings support: a) the applicability of the SMA definition as a construct/condition naturally occurring based on an underpinning network cluster of behaviours; b) a distinct association between SMA symptoms and distress behaviours related to depression, anxiety and stress, which advocates the separate classification of SMA as a psychopathological condition and; c) the role of mood-modification drives and functional impairment/conflicts with others as the connecting/linking points with stress/anxiety behaviours in the formation of SMA behaviours. Accordingly, results pose three significant taxonomic, assessment and prevention/intervention implications. Firstly, the consideration of SMA as a distinct diagnostic category is strengthened. Secondly, assessment of comorbid stress and anxiety manifestations appears to require priority when addressing clients presenting with problematic social media usage. Thirdly, though individuals of different ages and sexes tend to use social media in different ways, and thus likely experience SMA in different fashions, the effects of age and sex on SMA symptoms and their relationship with distress was not explored. This represents an important and interesting area of future study that deserves to be examined.

## Data Availability

The data and materials used in this study are available in this link https://github.com/Vas08011980/SNSNETWORK/blob/main/html.Rmd
